# Contribution of *VEGF* polymorphism rs3025020 to short stature and hypertension in elderly Japanese individuals: a cross-sectional study

**DOI:** 10.1186/s40101-021-00253-1

**Published:** 2021-02-04

**Authors:** Yuji Shimizu, Hirotomo Yamanashi, Shin-Ya Kawashiri, Kenichi Nobusue, Fumiaki Nonaka, Yuko Noguchi, Yukiko Honda, Kazuhiko Arima, Yasuyo Abe, Yasuhiro Nagata, Takahiro Maeda

**Affiliations:** 1 Department of Community Medicine, Nagasaki University Graduate School of Biomedical Sciences, Nagasaki, Japan; 2 Department of Cardiovascular Disease Prevention, Osaka Center for Cancer and Cardiovascular Diseases Prevention, Osaka, Japan; 3 Department of General Medicine, Nagasaki University Graduate School of Biomedical Sciences, Nagasaki, Japan; 4 Department of Islands and Community Medicine, Nagasaki University Graduate School of Biomedical Sciences, Nagasaki, Japan; 5 Department of Public Health, Nagasaki University Graduate School of Biomedical Sciences, Nagasaki, Japan

**Keywords:** Height, Hypertension, rs3025020, Short stature, VEGF

## Abstract

**Background:**

Recently, short stature has been revealed to be positively associated with hypertension, possibly because this indicates lower activity of vascular maintenance, such as angiogenesis. Vascular endothelial growth factor (VEGF) polymorphism (rs3025020) plays an important role in the progression of angiogenesis and may be associated with both hypertension and hypertension-associated short stature.

**Methods:**

A cross-sectional study of 1377 elderly Japanese individuals aged 60–89 years was conducted. Short stature was defined as the lowest tertile of height (< 160.8 cm for men and < 148.7 cm for women). Hypertension was defined as systolic blood pressure ≥ 140 mmHg and/or diastolic blood pressure ≥ 90 mmHg and/or antihypertensive medication use.

**Results:**

Independent of known cardiovascular risk factors, short stature was found to be positively associated with hypertension; the fully adjusted odds ratio (OR) and 95% confidence interval (CI) for hypertension were 1.51 (1.17, 1.96). With the reference group of carriers of the major allele of rs3025020, TT-homozygotes showed significantly lower OR for hypertension and short stature; the fully adjusted ORs (and 95% CIs) were 0.60 (0.41, 0.90) for hypertension and 0.59 (0.38, 0.91) for short stature, respectively.

**Conclusions:**

Angiogenesis-related genetic factor (rs3025020) is associated with hypertension and short stature, whereas short stature is positively associated with hypertension. Further investigation is necessary in this regard; the capacity for angiogenesis might partly explain the mechanism underlying the inverse association between height and hypertension.

## Introduction

Recent studies have reported an inverse association between height and hypertension [1, 2]. However, the mechanisms underlying this inverse association have not yet been clarified.

Hematopoietic stem cells (CD34-positive cells) contribute to maintaining micro-circulation, partly by inducing angiogenesis [3]. Previously, we found that lower levels of circulating CD34-positive cells could elevate the risk of hypertension among elderly men [4, 5]. Since height could act as a factor for CD34-positive cell production in the elderly [6–8], the risk of hypertension for short stature might be caused by disruption of microcirculation associated with a deficiency of angiogenesis.

Vascular endothelial growth factor (VEGF) is a proangiogenic factor. Inhibition of VEGF causes hypertension [9]. The minor allele (T) of the VEGF polymorphism rs3025020 is reported to be positively associated with serum levels of VEGF [10] and therefore could play an important role in the association between short stature and hypertension.

Although hematopoietic activity suitably explains the association between short stature and hypertension [4–8], the genetic factor involved in this association has not been identified. Since understanding these genetic associations may help elucidate the mechanism underlying the association between hypertension and short stature, we conducted a cross-sectional study of 1377 elderly Japanese individuals aged 60–89 years who participated in an annual health checkup in 2017.

## Material and methods

### Study population

The methods that relate to present risk surveys, including genetic factors, have been previously described [11–13].

The study population comprised 1388 individuals (501 men and 887 women) aged 60–89 years, from Goto city, located in the western part of Japan, who participated in an annual health checkup conducted by the local government and directed by the Ministry of Health, Labor and Welfare in Japan in 2017. Participants without serum data (*n* = 11) were excluded from the analysis. Finally, 1377 elderly Japanese individuals (498 men and 879 women), with a mean age of 72.8 (standard deviation [SD]: 7.1), were enrolled in the study.

### Data collection and laboratory measurements

Trained interviewers obtained the medical history and habitual status of each participant. Body weight and height were measured using an automatic body composition analyzer (BF-220; Tanita, Tokyo, Japan), from which the body mass index (BMI; kg/m^2^) was calculated.

Fasting blood samples were collected. Concentrations of triglyceride (TG), HDL-cholesterol (HDLc), hemoglobin A1c (HbA1c), and creatinine were measured using standard laboratory procedures. All measurements were performed by SRL, Inc. (Tokyo, Japan). The glomerular filtration rate (GFR) was estimated using a recently adapted established method introduced by a working group of the Japanese Chronic Kidney Disease Initiative [14], which yielded an estimate of GFR (ml/min/1.73 m^2^) = 194 × (serum creatinine [enzyme method])^−1.094^ × (age)^−0.287^ × (0.739 for women).

Genomic DNA was extracted from 2 mL of whole peripheral blood using Gene Prep Star NA-480 (Kurabo Industries Ltd., Osaka, Japan), and genotyping of single-nucleotide polymorphism (SNP) rs3025020 was conducted using TaqMan assays and a LightCycler 480 thermal cycling platform (Roche Diagnostics, Basel, Switzerland).

### Statistical analysis

Characteristics of the study population by rs3025020 genotype are expressed as mean ± SD, except for prevalence of antihypertensive medication use and current smoker and daily drinker status. Significant differences involving rs3025020 genotypes were evaluated using analysis of variance.

Logistic regression models were used to calculate odds ratios (ORs) and 95% confidence intervals (CIs) to determine associations between hypertension and short stature, between rs3025020 genotypes and hypertension, and between rs3025020 genotypes and short stature.

Two adjustment models were used. The first model (model 1) was adjusted only for sex and age. The second model (model 2) further included potential confounding factors known as cardiovascular risk factors, namely, BMI (kg/m^2^), smoking status (current, former, or never), drinking status (non, often, or daily), TG (mg/dL), HDLc (mg/dL), HbA1c (%), and GFR (mL/min/1.73 m^2^).

We also evaluated the sex-adjusted values of height and the prevalence of short stature associated with the two categories of the rs3025020 genotype (carriers of the major allele and homozygous for the minor allele) using analysis of covariance.

The definition of short stature was the sex-specific lowest tertiles of height values (< 160.8 cm for men and < 148.7 cm for women). Hypertension was defined as systolic blood pressure ≥ 140 mmHg and/or diastolic blood pressure ≥ 90 mmHg and/or current antihypertensive medication use.

All statistical analyses were performed using the SAS system for Windows (v.9.4; SAS Inc., Cary, NC, USA). Values of *p* < 0.05 were considered statistically significant.

## Results

### Study population characteristics

Among the study population, the number of individuals with each rs3025020 genotype was 734 for CC-homozygotes, 515 for heterozygotes, and 128 for TT-homozygotes.

Table 1 presents the characteristics of the study population in relation to the rs3025020 genotype. The minor rs3025020 allele (T) was inversely associated with systolic blood pressure. Sex-specific height levels (mean ± SD) and (number of participants [*n*]) for each category among men were 163.7 ± 6.2 cm (*n* = 279) for homozygotes (C/C), 163.2 ± 6.2 cm (*n* = 174) for heterozygotes (C/T), and 162.6 ± 5.9 cm (*n* = 45) for homozygotes (T/T) *(*p = 0.487), and the corresponding values among women were 150.6 ± 5.9 cm (*n* = 455), 150.9 ± 5.9 cm (*n* = 341), and 152.6 ± 5.9 cm (*n* = 83) (*p* = 0.015), respectively.

**Table 1:** Characteristics of study population by rs3025020 genotype

	rs3025020			*p* value
	(C/C)	(C/T)	(T/T)	
No. of participants	734	515	128	
Male, %	38.0	33.8	35.2	0.301
Age	72.5 ± 7.0	73.0 ± 7.3	73.1 ± 6.9	0.447
Height, cm	155.6 ± 8.8	155.0 ± 8.3	156.1 ± 7.6	0.318
Systolic blood pressure, mmHg	135 ± 17	135 ± 18	131 ± 16	0.044
Diastolic blood pressure, mmHg	74 ± 10	74 ± 11	73 ± 10	0.369
Anti-hypertensive medication, %	43.5	42.3	36.7	0.363
Current smoker, %	17.3	15.3	14.8	0.944
Daily drinker, %	7.6	8.2	7.8	0.582
Body mass index (BMI), kg/m^2^	22.6 ± 3.3	22.7 ± 3.1	22.6 ± 3.3	0.909
Triglycerides (TG), mg/dL	106 ± 57	104 ± 52	95 ± 59	0.148
HDL-cholesterol (HDLc), mg/dL	62 ± 15	62 ± 16	64 ± 16	0.377
Hemoglobin A1c (HbA1c), %	5.8 ± 0.5	5.8 ± 0.6	5.7 ± 0.5	0.679
Glomerular filtration rate (GFR), mL/min/1.73 m^2^	68.8 ± 14.3	69.0 ± 14.0	69.2 ± 14.2	0.934

Values: mean ± standard deviation

### Association between short stature and hypertension

Among elderly Japanese individuals, short stature was found to be positively correlated with hypertension. This association was unaffected even after adjusting for known cardiovascular risk factors (Table 2).

**Table 2:** Odds ratios (ORs) and 95% confidence intervals (CIs) for hypertension in relation to short stature

	Short stature		*p* value
	(−)	(+)	
No. of participants	919	458	
No. of cases (%)	503 (54.7)	311 (67.9)	
Model 1	1	1.40 (1.10, 1.79)	0.007
Model 2	1	1.51 (1.17, 1.96)	0.002

Model 1: adjusted only for sex and age. Model 2: adjusted further for body mass index (BMI), smoking status, drinking status, triglycerides, HDL-cholesterol, HbA1c, and glomerular filtration rate (GFR). Short stature was defined as the lowest tertile of height (< 160.8 cm for men and < 148.7 cm for women). Hypertension was defined as systolic blood pressure ≥ 140 mmHg and/or diastolic blood pressure ≥ 90 mmHg and/or taking anti-hypertensive medication

### Association between rs3025020 genotype and hypertension

Independent of known cardiovascular risk factors, the minor allele (T) of rs3025020 was significantly inversely associated with hypertension. When we defined a reference group of carriers of the major allele (C/C and C/T), homozygotes of the minor allele (T/T) exhibited significantly lower ORs for hypertension (Table 3).

**Table 3:** Odds ratios (ORs) and 95% confidence intervals (CIs) for hypertension in relation to rs3025020 genotypes

	rs3025020			*p* value	Minor allele (T)
	(C/C)	(C/T)	(T/T)		
No. of participants	734	515	128		
No. of cases (%)	442 (60.2)	309 (60.0)	63 (49.2)		
Model 1	1		0.61 (0.42, 0.89)	0.010	0.84 (0.71, 0.997)
	1	0.96 (0.76, 1.22)	0.61 (0.41, 0.89)	0.046	
Model 2	1		0.60 (0.41, 0.90)	0.012	0.83 (0.69, 0.98)
	1	0.93 (0.73, 1.19)	0.59 (0.39, 0.89)	0.032	

Model 1: adjusted only for sex and age. Model 2: adjusted further for body mass index (BMI), smoking status, drinking status, triglycerides, HDL-cholesterol, HbA1c, and glomerular filtration rate (GFR). Hypertension was defined as systolic blood pressure ≥ 140 mmHg and/or diastolic blood pressure ≥ 90 mmHg and/or taking anti-hypertensive medication

### Association of sex-adjusted values of height and the prevalence of short stature for the rs3025020 genotype (carriers of the major allele and homozygous for the minor allele)

Although no significant correlation between the sex-adjusted values of height and two categories of rs3025020 (carriers of the major allele and homozygous for the minor allele) was observed, a significantly low prevalence of short stature was observed in individuals who were homozygous for the minor allele compared with those who were carriers of the major allele. The sex-adjusted values of height (least squared value ± standard error) and prevalence of short stature (least squared value) were 155.3 ± 0.2 cm and 34.1% for carriers of the major allele and 156.3 ± 0.5 cm and 25.0% for those homozygous for the minor allele (*p* = 0.090 and 0.038), respectively.

### Association between rs3025020 genotype and short stature

Table 4 illustrates the association between rs3025020 genotype and short stature. Even though no significant association between the minor allele of rs3025020 and short stature was observed, an inverse tendency involving the two was noted. When we set a reference group of carriers of the major allele, homozygotes for the minor allele showed significantly lower ORs for short stature. This association was unchanged even after adjustment for known cardiovascular risk factors.

**Table 4:** Odds ratios (ORs) and 95% confidence intervals (CIs) for short stature in relation to rs3025020 genotypes

	rs3025020			*p* value	Minor allele (T)
	(C/C)	(C/T)	(T/T)		
No. of participants	734	515	128		
No. of cases (%)	246 (33.5)	180 (35.0)	32 (25.0)		
Model 1	1		0.61 (0.40, 0.94)	0.024	0.88 (0.73, 1.05)
	1	1.03 (0.80, 1.32)	0.62 (0.40, 0.96)	0.152	
Model 2	1		0.59 (0.38, 0.91)	0.017	0.86 (0.72, 1.03)
	1	1.01 (0.79, 1.30)	0.59 (0.36, 0.93)	0.103	

Model 1: adjusted only for sex and age. Model 2: adjusted further for body mass index (BMI), smoking status, drinking status, triglycerides, HDL-cholesterol, HbA1c, and glomerular filtration rate (GFR). Short stature was defined as the lowest tertile of height (< 160.8 cm for men and < 148.7 cm for women)

To test the sensitivity, we re-performed the main analyses using the sex-specific model and obtained essentially the same associations. For the association between short stature and hypertension, the age-adjusted ORs (and 95% CIs) were 1.51 (1.01, 2.25) for men and 1.29 (0.94, 1.77) for women. The age-adjusted value of ORs (and 95% CIs) of short stature with the rs3025020 genotype (using carriers of the major allele as a reference group) for homozygotes for the minor allele were 0.95 (0.49, 1.82) for men and 0.46 (0.25, 0.81) for women. The age-adjusted value of ORs (and 95% CIs) of hypertension with the rs3025020 genotype (using carriers of the major allele as a reference group) for homozygotes for the minor allele were 0.52 (0.28, 0.97) for men and 0.68 (0.42, 1.09) for women.

## Discussion

In this study, we demonstrated that homozygotes for the minor allele of the VEGF rs3025020 were found to be inversely associated with hypertension and short stature when compared with a reference group of carriers of the major allele; short stature was positively associated with hypertension.

Height is reported to be inversely associated with hypertension [1, 2], which is consistent with our present results. However, the possible mechanisms underlying this association have not yet been clarified.

Disruption of microcirculation, which elevates peripheral vascular resistance, can cause hypertension [15]. Angiogenesis is one of the most important processes for maintaining microcirculation. Previously, we reported that height is positively associated with levels of hematopoietic stem cell (CD34-positive cells), which contribute to angiogenesis among elderly Japanese men [3, 6–8]. Since hematopoietic bone marrow activity declines with age [16–18] and absolute bone marrow volume is positively associated with height, this positive association between height and CD34-positive cells could be caused by age-related reduction in bone marrow activity. Therefore, short stature is a risk factor for age-related reduction of bone marrow that causes tissue hypoxia. In this mechanism, VEGF expression levels could be elevated in participants with short stature, as tissue hypoxia associated with lower angiogenic activity might increase VEGF abundance [19].

However, in the present study, the minor allele homozygote (T/T) participants showed a significantly lower OR for short stature. Since the minor rs3025020 allele is reported to be positively associated with serum levels of VEGF [10], age-related reduction of bone marrow could not explain this association.

Height is generally considered a marker of social and physical conditions during childhood and adolescence [20–24], although genetic factors may also influence physical conditions during childhood (adolescence). During the growth period, VEGF plays an important role in skeletal development by stimulating angiogenesis and endochondral ossification [25, 26]. Therefore, genetic characteristics that exhibit beneficial effects on elevating VEGF [10] could result in a comparatively higher growth rate in childhood when necessary, leading to non-short stature in adulthood. However, this beneficial effect of rs3025020 on height development is only supportive; this genetic characteristic may help prevent delayed development of height in childhood, resulting in a lower prevalence of short stature but not an accelerated development of height. In the present study, homozygotes for the minor allele exhibited a significantly lower prevalence of short stature than carriers of the major allele; however, there was no significant difference between the two groups for sex-adjusted values of height. Height is affected by various genetic and environmental factors, and unknown factors might influence the observed associations. Furthermore, aging is associated with height loss [27]; in line with this notion, subjects with short stature (75.3 ± 7.2) in the present study were significantly older than those without short stature (71.5 ± 6.6, *p* < 0.001). Thus, age-related height loss may have influenced the present results. However, in the present study, no significant association was observed between the minor rs3025020 allele (T) and age. Therefore, the influence of age-related height loss is expected to be limited. Notably, we observed a significant association between rs3025020 and short stature even after adjusting for age.

Additionally, hypoxia contributes to functional degeneration during the aging process [28]; hypoxia is considered a well-known cause of hypertension [29]. Since homozygotes (T/T) of the rs3025020 minor allele may exert beneficial effects in terms of compensation for hypoxia by inducing angiogenesis, such individuals may also experience beneficial effects such as the absence of hypertension in later life.

However, we believe that rs3025020 is not a determinant factor, but one of several factors contributing to the association between hypertension and short stature. We found a significant positive association between hypertension and short stature when we limited this analysis to major allele homozygosity (C/C), where the fully adjusted OR (and 95% CI) was 1.56 (1.09, 2.22). Age-related hypoxia increases the possibility of angiogenesis. This genetic characteristic might influence the magnitude of response to hypoxia, which stimulates angiogenesis [30]. A previous Spanish study reported that T-alleles in rs3025020 were less frequent in patients with chronic obstructive pulmonary disease (COPD) and were associated with a lower risk of developing the disease [31]. This study partly supports our hypothesis that the present genetic characteristics may influence the magnitude of response to hypoxia because T-alleles in rs3025020 show beneficial effects in preventing hypoxia related to COPD. Therefore, involvement of rs3025020 could partly explain the association between hypertension and short stature. Moreover, rs3025020 and rs3025039 might play an important role in the association between hypertension and short stature. The minor allele of rs3025020 is positively associated with VEGF levels, whereas rs3025039 is inversely associated with VEGF levels [10]. Therefore, further investigation using rs3025039 data should be performed in future studies. VEGF is important for bone formation and has also been associated with osteoporosis [32]. Since short stature in elderly is likely to be associated with osteoporosis and essential hypertension can reduce bone mineral density [33], osteoporosis may have influenced the present results. However, no significant association was observed between the minor rs3025020 allele (T) and age. Therefore, we believe that the influence of osteoporosis was likely limited. Further investigations in the context of osteoporosis are necessary.

The results of the present study indicate that genetic factors that may exert beneficial effects on skeletal development during childhood could also prove to be beneficial in preventing hypertension in later life. These associations may partly explain the mechanism underlying the association between hypertension and short stature.

Potential limitations of the present study warrant consideration. Due to the shortage of serum samples, we could not evaluate serum VEGF levels, which might have influenced the present results. Further investigations surrounding these data are necessary. Owing to the limited sample size in this study, precise sex-specific analyses, especially for men, could not be performed. For women, a significant positive association between the minor rs3025020 allele (T) and height and a significant inverse association between the minor rs3025020 allele (T) and short stature were observed. Notably, for men, an inverse tendency (though insignificant) was observed between the minor rs3025020 allele (T) and height. However, the OR of subjects with short stature in the context of the rs3025020 genotype (using carriers of the major allele as a reference group) for homozygotes (T/T) was 0.95 (0.49, 1.82), which is below 1. We hypothesize that this genetic characteristic may lead to a lower prevalence of short stature but not of accelerated growth. Further studies based on larger study populations are warranted to clarify this.

## Conclusions

In conclusion, with reference to the group of carriers of the major allele, homozygosity of the minor allele of rs3025020 was found to be inversely associated with hypertension and short stature, and short stature was found to be positively associated with hypertension. The capacity for angiogenesis indicated using the genetic factor (rs3025020) may partly explain the mechanism underlying the inverse association between height and hypertension.

## Data Availability

We cannot publicly provide individual data due to participant confidentiality, according to ethical guidelines in Japan. Additionally, obtaining informed consent does not include a provision for publicity sharing data. Qualifying researchers may apply to access a minimal dataset by contacting Prof Takahiro Maeda, Principal Investigator, at tmaeda@nagasaki-u.ac.jp. Please contact the office of data management at ritouken@vc.fctv-net.jp. Information for where the data request is also available at http://www.med.nagasaki-u.ac.jp/cm/.

## Abbreviations

VEGF: Vascular endothelial growth factor
OR: Odds ratio
CI: Confidence interval
SD: Standard deviation
BMI: Body mass index
TG: Triglyceride
HDLc: HDL-cholesterol
HbA1c: Hemoglobin A1c
GFR: Glomerular filtration rate
n: Number of participants
COPD: Chronic obstructive pulmonary disease
